# Does My Face FIT?: A Face Image Task Reveals Structure and Distortions of Facial Feature Representation

**DOI:** 10.1371/journal.pone.0076805

**Published:** 2013-10-09

**Authors:** Christina T. Fuentes, Catarina Runa, Xenxo Alvarez Blanco, Verónica Orvalho, Patrick Haggard

**Affiliations:** 1 Institute of Cognitive Neuroscience, University College London, London, United Kingdom; 2 Instituto de Telecomunicações, Porto Interactive Center, Universidade do Porto, Porto, Portugal; University G. d'Annunzio, Italy

## Abstract

Despite extensive research on face perception, few studies have investigated individuals’ knowledge about the physical features of their own face. In this study, 50 participants indicated the location of key features of their own face, relative to an anchor point corresponding to the tip of the nose, and the results were compared to the true location of the same individual’s features from a standardised photograph. Horizontal and vertical errors were analysed separately. An overall bias to underestimate vertical distances revealed a distorted face representation, with reduced face height. Factor analyses were used to identify separable subconfigurations of facial features with correlated localisation errors. Independent representations of upper and lower facial features emerged from the data pattern. The major source of variation across individuals was in representation of face shape, with a spectrum from tall/thin to short/wide representation. Visual identification of one’s own face is excellent, and facial features are routinely used for establishing personal identity. However, our results show that spatial knowledge of one’s own face is remarkably poor, suggesting that face representation may not contribute strongly to self-awareness.

##  Introduction

Face perception is a central topic in modern psychology. The field has overwhelmingly used visual stimuli and focussed on face recognition, even when considering perception of one’s own face [1]. People see their own face only rarely – vanishingly rarely until the recent ready availability of mirrors. Nevertheless, several studies indicate a specific mechanism involved in recognising one’s own face (e.g., [2], see also 3 for a review). Much of this literature has on focussed sensitivity to facial symmetry and its relation to effects of mirrors [4,5], and cerebral hemispheric specialisation [6]. Many visual face recognition studies suggest a superior and accurate visual representation of one’s own face [3]. However, the persistence of this advantage even when faces are inverted suggests that it relies on local rather than configural processing [7].

In general, the self-face visual recognition literature cannot readily distinguish between self-face processing based on familiarity with a visual image of one’s own face suitable for template matching, or based on structural knowledge about what one’s face is like (i.e., a face image or a hypothetical stored representation containing information about the positions of facial features relative to one another, akin to the body structural description [8]. Here we largely remove the visual recognition aspect of self-face processing to focus on the latter, structural representation aspect. Only one study has investigated somatosensory self-face perception [9], and found generally poor performance. Therefore, it remains unclear what people know about their own facial structure, and how this knowledge is stored and represented independent of a specific visual stimulus.

We recently developed tasks for studying the sensed position of body parts (Longo and Haggard, 2012), and stored models of one’s own body [10,11]. These representations both showed systematic patterns of distortion, which potentially indicate how spatial information about bodies is represented and stored in the brain. Here we report results on representation of one’s own facial features using a method that does involve visual recognition. We show, first, that people make large errors in locating their own facial features, particularly underestimating face height. Second, we show through factor analysis that the representation of facial feature locations follows a characteristic structure. The patterns of localisation errors showed covariance across specific subsets of features, which may be relevant to identifying the organisation of face representation at a supra-featural, or configural level. The overall structure of face representations implies an important distortion of face shape. Our work provides a novel and systematic approach to a classic question of Gestalt psychology: how are configurations of multiple features represented in the brain as a composite pattern? Our results may also be relevant to the considerable concern regarding one’s own facial structure and appearance in some individuals and cultures.

## Methods

### Ethics Statement

All participants gave informed written consent. All experiments were approved by the local ethics committee at University College London.

**Figure 1:**
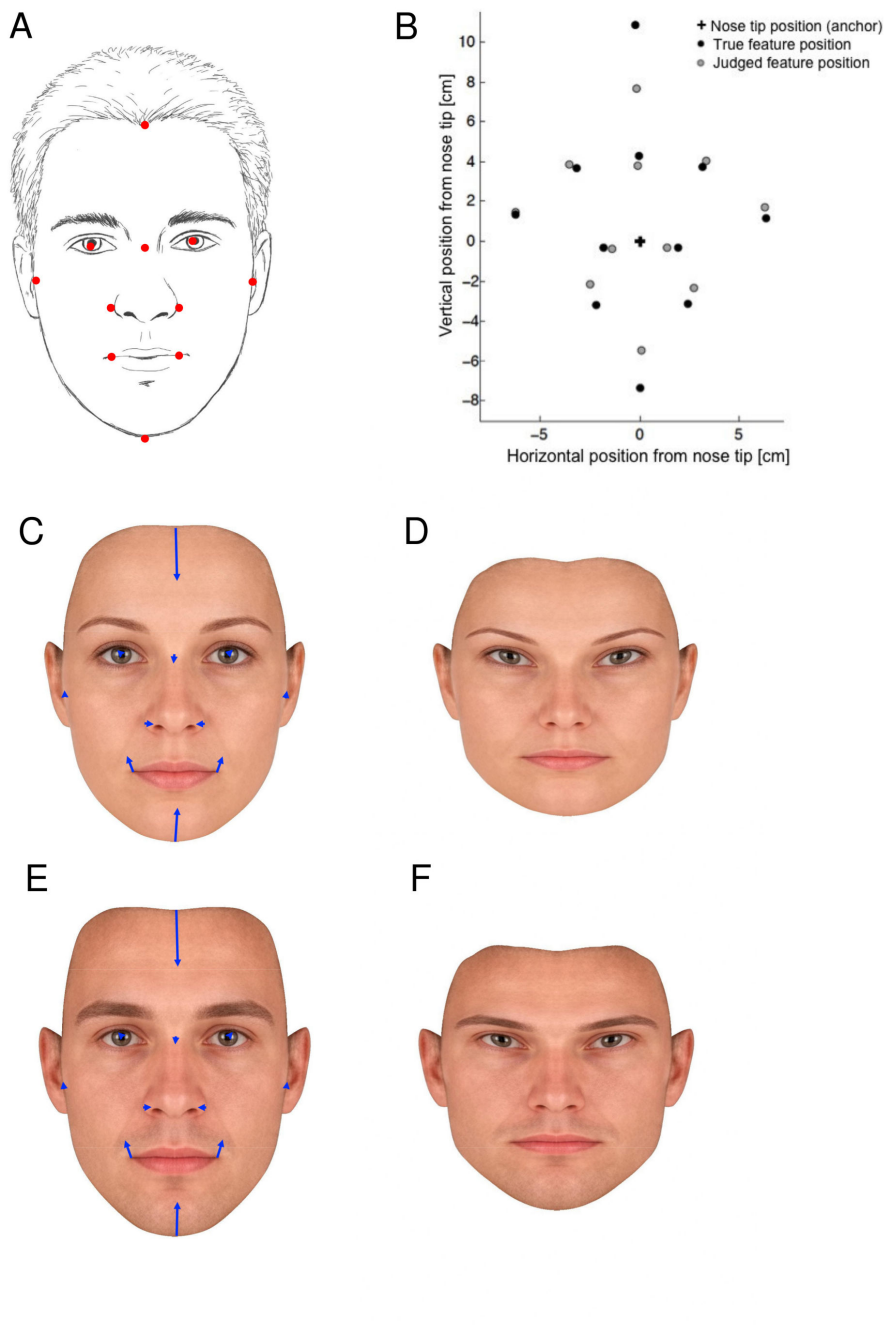
Biases in face representation. A Schematic of feature locations used to instruct participants. B. Actual and mean represented locations. C, Average of 50 female faces reproduced with permission from www.perceptionlab.com. Blue arrows indicate mean judgement error for each feature. D. Average female face adjusted according to the mean represented locations of our participants. E, F: as for C, D with average of 50 male faces.

Participants were seated in front of a computer screen in portrait orientation (Dell model 2007 WFPb, measuring 43.5 cm vertical, 27.5 cm horizontal) which displayed only a small central dot. The position of the dot on the screen was randomised across trials. Participants were instructed to imagine their own face projected frontally, life-size on the screen, with the tip of the nose located at the dot. They used a mouse to indicate the locations corresponding to 11 landmark facial features The figure reproduced as Figure 1A was shown to participants before the experiment to indicate the exact anatomical landmarks intended. Before each trial, a text label (e.g., “botton of chin”, “centre of left eye”) briefly appeared centrally on the screen. Environmental lighting was controlled so that they could not see any reflection of their face on the screen. Each landmark was judged five times in a random order. To quantify errors in perceived position of facial features, responses were later compared to the actual locations of those landmarks, obtained by taking a photograph under standardized conditions and rendering it at life-size on the same screen. The average horizontal (x) and vertical (y) error for attempts to locate each facial landmark were calculated.

Fifty participants (24 female, average age 25 years) took part. The x data from left-sided landmarks (ears, nose and mouth edges, eyes) was reflected in the midline, and averaged with the corresponding right-sided landmark. This imposed an assumption of facial symmetry, but reduced the number of dependent variables and avoided possible confusion regarding the terms *left* and *right* in the context of the task. By analysing the pattern of errors, we aimed to investigate the internal stored representation of one’s own face.

Finally, a subset of 10 participants were asked to attend for a second session, in which the screen was rotated to landscape mode.

## Results

The average error vectors are shown superimposed on a schematic face in Figure 1. They reveal large overall biases in locating facial landmarks. The anatomical structure of the face is very different in the horizontal and vertical dimensions. The horizontal dimension is characterised by symmetry and homology, while the vertical dimension lacks both these attributes. Therefore, we expected different patterns of error in the X and Y dimensions, and accordingly analysed each dimension separately. In the horizontal dimension, mouth and eye width are overestimated, while nose width is underestimated. In the vertical dimension, the hairline is represented as lower, and the chin as higher, than their true locations, suggesting that the face is represented as shorter than its true height. No simple geometric distortion can explain the *overall* pattern of biases: for example, the compression of face height may appear to be a regression of judgement towards the mean defined by the anchor point on the nose tip. However, eye and ear vertical positions appear to be unaffected by this bias, and the bias is absent in the horizontal dimension, suggesting it is not simply a matter of eccentricity. Moreover, Bonferroni-corrected testing showed significant biases for some facial features close to the anchor point, but not for those farther away (table 1).

**Table 1:** Average localisation errors for each feature in cm.

Part	Mean Horizontal Error (cm) (SD)	Mean Vertical Error (cm) (SD)
Hairline	-0.0875 (0.2989)	**-3.1533 (1.8734)**
Chin	-0.0640 (0.3028)	**1.8987 (1.6650)**
Ear	0.0396 (1.5981)	0.3534 (1.7092)
Nose Bridge	**0.0735 (0.1401)**	-0.4734 (1.3835)
Nose	**0.4995 (0.6141)**	-0.0246 (0.5963)
Mouth	-0.3170 (1.0228)	**0.9060 (0.9188)**
Eyes	-0.2510 (1.0588)	0.2509 (1.4582)

Values that are significantly different from 0 (p<.05, after Bonferroni correction for 7 tests) are shown in **bold type**.

In the ten participants who performed the task with the screen in portrait and landscape mode, we found no effects of screen orientation on judgement error, and no interaction between screen orientation and feature judged, in either X or Y dimensions (all F<1, all p>0.60).

To investigate the underlying *structure* of the face representation shown in Figure 1, we applied separate factor analyses to x and y judgement errors (tables S1 and S2). The ratio of measurements-to-cases falls within the guideline range for exploratory factor analysis [12]. Principal components were extracted, and varimax rotated. Factors with eigenvalues over 1 were retained (table 2 and Figure S1). 

**Table 2:** Factor scores for horizontal X and vertical Y components.

Factor	X1	X^2^	X3	Y1	Y2
Eigenvalue	2.75	1.70	1.05	3.09	1.84
Variance proportion	39%	24%	15%	44%	26%
Hairline	-0.00134	0.91808	-0.08091	0.86393	-0.14580
Chin	-0.02570	-0.01205	0.97501	-0.45231	0.76155
Nose bridge	0.09507	0.89391	0.07555	0.89044	-0.14270
Nose edge	0.66612	-0.24710	-0.11494	0.21907	0.77971
Mouth	0.88904	0.09058	-0.14932	-0.17919	0.92808
Eye	0.90951	0.14324	0.01498	0.92128	-0.02805
Ear	0.78575	0.14074	0.23051	0.32930	0.24252

Only factors with eigenvalues over 1 are shown.

For horizontal errors, we identified three retainable factors, which we label X1, X^2^, X3 for convenience, corresponding to the principal, independent sources of variability in horizontal judgement errors for facial features. The first factor (X1) suggested a tendency to expand facial width outward from the midline. It loaded strongly and roughly equally on all lateralised structures (eye, mouth, ear, nose), but not on midline structures (centre of hairline, bridge of nose, chin). The second factor (X^2^) suggested lateral distortion of the upper face. It loaded largely on the hairline and nose bridge. The third factor (X3) suggested lateral distortion of the lower face, loading almost exclusively on the chin. For analysis of vertical errors, only two factors were retained. The first (Y1) loaded strongly on upper face structures (eyes), including midline structures (nose bridge, hairline), but with some modest negative loading on the chin. This factor suggested a vertical expansion of the face from its centre. The loadings of the second factor (Y2) on lower face structures (mouth, nose edges, chin) suggest a vertical shift confined to the lower face.

We investigated the relation between the factors underlying face representation and our participants’ actual facial features, as measured from photos. Since factor X1 was interpreted as the width of the face, we correlated scores on this factor with the actual ear-to-ear distance. Since factor Y1 was interpreted as the vertical height of the face, we correlated it with the actual hairline-to-chin distance. We found no associations between represented and actual facial dimensions (r=-0.036 NS and 0.016 NS, respectively).

These factor solutions carry important information about the internal structure of horizontal and vertical face representation. Factors X1, X^2^, Y1 and Y2 all loaded on more than one facial feature. The loading patterns suggest complexes of two or more individual features that group together, and which covary across the face representations of different individuals. By this means, we could identify separable representations of lateral and midline horizontal facial features, and separable representations of upper and lower face vertical structure. The effects of varying each factor on an average face are shown as vectors in Figure S1, and pictorially in Figure S2.

We also investigated the overall geometry of face representation by seeking an inter-domain association between factors affecting horizontal and vertical errors. We used canonical correlation to identify the principal associations between our horizontal factors (X1, X^2^) and vertical factors (Y1, Y2).

**Figure 2:**
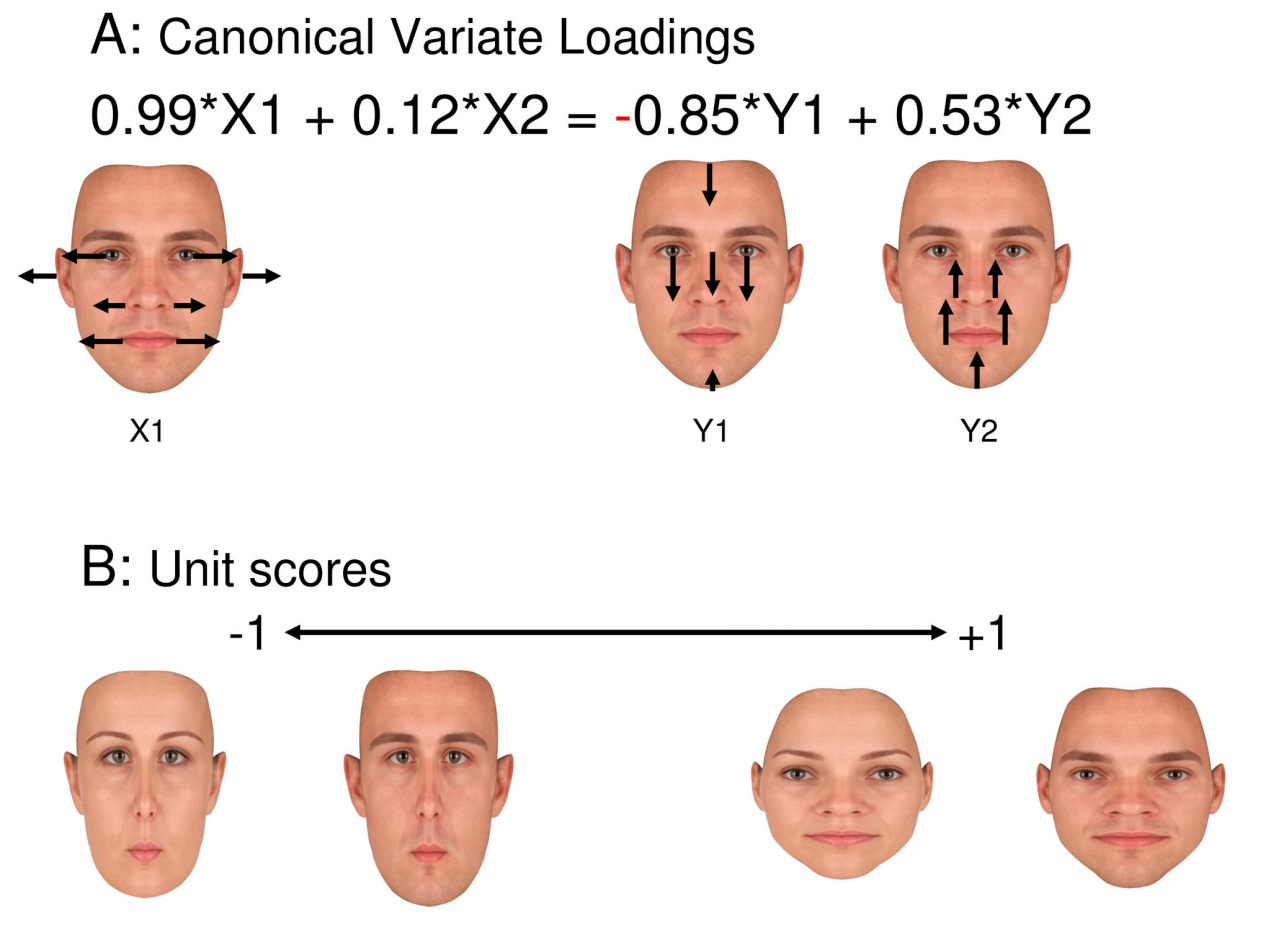
Association between horizontal and vertical distortion factors demonstrates variation in representation of face shape across individuals. Results of a canonical correlation between the horizontal (X1,X2) and vertical (Y1,Y2) factors. A. Vectors showing the principal feature loadings (>0.4 or <-0.4) of the factors, adjusted by the coefficients indicating important (>0.4 or <-0.4) contributions to the canonical variate. The vector lengths are shown at 4x the actual values for visual clarity. Note the negative sign for Y1 coefficient. B. Average female and male faces implied by a low and high score on the canonical variate. Note that the canonical variate separates long and thin from short and wide face representations.

The first canonical variate accounted for 48.5% of the variance between the horizontal and vertical factors and was highly significant (Wilks’ Lambda 0.506, approximated by F(4,92)=9.34, p<.001). The standardised weights showed that the canonical variate related X1 (weighting 0.99) negatively to Y1 (-0.85) and positively, though less strongly, to Y2 (0.53). In contrast, factor X^2^ made little contribution to this inter-domain association (weighting 0.12), suggesting that it constituted an independent aspect of facial structure. The combination of weightings in the first canonical variate is readily interpretable as face aspect ratio, or 2D shape. The lateral shift of eyes, mouth edges, ears and nose captured by factor X1 was associated with a downward shift of the hairline and nose-bridge (captured by Y1), and some upward shift of the mouth, nose edges and chin (captured by Y2). That is, the lateral expansion of the face was strongly associated with a vertical compression of towards the face centre, suggesting that the face aspect ratio is the major structural principle of face representation. The second canonical variate explained only 1.7% of the shared variance between factors, and was far from significant (p=0.37). Factor X3 was excluded from the inter-domain analysis, as its loading was largely confined to a single feature. However, re-running the analysis with this factor included had only small effects on weightings of inter-domain association and did not change the pattern of inference. Figure 2A shows the vectors associated with the major loadings (>0.4) of each factor, adjusted by the factor’s weighting in the canonical variate. Figure 2B shows the face images implied by a positive and negative unit score on the canonical variate.

## Discussion

We have developed a new method to investigated stored knowledge about the “face image”, or structural arrangement of one’s own facial features. Importantly, this method allows the structural description of the face to be investigated independent of visual recognition.

Analyses of errors in locating facial landmarks relative to the tip of the nose suggested an internal representation or model of one’s own face, with characteristic structure. We first showed an overall bias to represent face shape as shorter than it really is. This bias was unrelated to the actual height and width of an individual’s face. Second, we showed that the most prominent signature of different individuals’ overall face representations is the extent to which they express a set of associated factors that code for tall/thin vs short/wide face representation. This recalls similar shape distortions for the position sense of the hand [13], and for the body image [10]. Since shape and size of body parts is not directly signalled by any somatosensory receptor [14], it may be unsurprising that face representation is non-veridical. However, our results show, for the first time, that errors in facial representation are not simply random noise, or regression to the mean, but have a systematic structure.

One striking component of this structure was the aspect ratio defined by facial features. We investigated horizontal and vertical structure of face representation in two independent analyses. We next investigated the association of these dimensions, and found that facial aspect ratio emerged as a prominent feature of the data pattern. Our data therefore provides strong and independent convergent evidence that aspect ratio is a major source of variation in face representation. Not only are people poor at estimating the shape of their own face (Figure 1), but the principal source of variation across individuals is in the biased representation of face shape.

A second clear component of face structure was the separation between upper and lower facial features. For most of the factors we extracted, we found that high loadings on the upper face were accompanied by low loadings on the lower face, or *vice versa*. This dissociation could reflect innervation by different branches of the trigeminal nerve, or it could reflect different functions of the upper face (gaze, attention) and lower face (speech, eating). In any case, our data confirm a fundamental division in face *representation*, as opposed to face perception, between upper and lower face.

Third, we found important misrepresentations of the lateral position of midline structures. Interestingly, these midline shifts occurred independently for the upper face (factor X^2^) and lower face (factor X3), providing further strong evidence for independent representation of upper and lower face, but this time from the orthogonal, horizontal dimension of representation. We note that factor X3 requires a more cautious interpretation, given the marginal eigenvalue and loading on a single feature (the chin). The two midline shift factors could be interpreted as forehead and mandibular asymmetry, respectively. The importance of symmetry in developmental and evolutionary biology is widely accepted [15], and fluctuating asymmetry is also thought to be used as a proxy for biological quality in mate selection [16]. Alternatively, our findings of may reflect brain functions underlying face representation, rather than sensitivity to body morphology. Neuroscientific studies suggest that the two cerebral hemispheres may play different roles in face perception [17]. Variation across individuals in such hemispheric specialization might also explain asymmetric representation of one’s own face.

Distortions in face representation have been widely reported in visual perception. For example, one study using adaptation procedures investigated aspect suggested that aspect ratio was a core component of face coding in the human brain [18]. However, those studies did not specifically test for *other* distortions of face coding, apart from shape, and could design only a limited range of stimuli to test dimensions of coding hypothesised a priori. In our approach, by contrast, the key dimensions of face coding emerge from the pattern of participants’ responses, rather than by experimenters’ choice of stimulus set.

### Configural processing

Models of face perception distinguish between information about individual facial features, and ‘holistic’ or ‘configural’ information about spacing between features [19]. Psychophysical studies, for example using the composite face effect, confirm that configural information plays an important role in face perception [20,21], and that this information is processed ‘holistically’. However, the structure of the underlying Gestalt or face configuration is not known. Most previous studies have focussed on spatial relations between facial features that are either hypothesised a priori, or motivated by general processing considerations independent of face perception. These include relations between the upper and lower and left and right facial features [17]. In contrast, the Face Image Task (FIT) provides a new, hypothesis-free method for investigating how multiple features are combined in configural representations, at least for representation of one’s own face.

In particular, our factor method extracted distinct sets of features whose representations tended to covary, even though we did not impose such a pattern of variation by designing our stimuli, and even though only one feature was ever judged at a time. This grouping of features was not simply defined by proximity (e.g., the edges of the nose grouped with the chin in factor Y2, not with the eyes, despite being closer to the latter than to the former). We suggest that such feature grouping may underlie configural face processing, and could provide a useful data-driven method for identifying what structural information is actually stored in the hypothesised configural representation. Configural processing might reflect precise representation of the spatial relations of features *within* a group, while spatial relations *between* groups of features might be less precisely represented. These findings generate testable predictions for future face-recognition experiments. For example, laterally shifting ears relative to eyes should be readily detectable, due to the common high loadings of these features on factor X1. But vertically shifting ears relative to eyes should be less detectable, since these features are not strongly grouped by any important factor.

### Perceptual and productive self-representation

Our results show that the structural knowledge about one’s own facial features is remarkably poor. This contrasts with numerous results in visual self-face recognition showing that self-face processing is remarkably good, and superior to processing of other faces (e.g., [7]). Our results suggest that the internal *representation* of the face is strongly and systematically distorted, but we have no difficulty in recognising much smaller distortions when *viewing* faces (Figure 1). This points to a dissociation between the processes of matching visual input to a perceptual template, and the processes of accessing structural representations directly for purposes of reproducing them. Artists often improve their face drawing skills by learning geometric rules regarding the spacing of facial features. This may be considered a transfer of training from perceptual representation to productive representation. Interestingly, this process is accompanied by strengthened representation of local featural detail in face perception, at the expense of holistic, configural processing [22,23]. Comparisons of self-face and other-face processing also suggest a dominance of local over configural information for one’s own face [24]. Our data suggest that configural information about one’s own face is also poorly represented because there are systematic biases in judgements about feature locations. Nevertheless, we found grouping of features in virtue of loading on a single factor. This suggests that some configural structure to face representation is present, albeit of limited accuracy.

In addition, our results offer a dramatic example of the asymmetry between fluent, automatic, stimulus-driven access to object representation, and the limited accessibility of such object representations to the kind of deliberate controlled processing involved in our task. Even our own face appears to be impenetrable to controlled cognition. It is well-known from memory research that recognition is superior to recall. In contrast, the everyday concept of self-awareness implies an opposite pattern. We do not need to recognise our thoughts and mental states as ours. Rather, a stable, persistent core self is held to be directly known, and to provide an origin for mental states, attitudes and actions. This account of the self has recently been questioned [25]. Our approach suggests that bodily self-knowledge is poor, even for elements such as the face, which may be important for personal identity. Therefore, if there is a stable core self underlying self-identity, knowledge about the physical structure of one’s own face does not appear to be strongly linked to it.

### Specificity

It is unclear whether the distortions reported here are specific to representing one’s own face, or indeed to faces as a category. Identifying suitable objects for a control task is problematic. The quality and quantity of experience we have with other people’s faces, and with non-face objects, is entirely different from the experience of our own face. Controlling for modality, familiarity, prototypicality and other relevant factors is therefore difficult. Further, the features of non-face objects cannot match those of faces in number, salience and configuration, almost by definition. Thus, the representation of information about faces cannot easily be compared to representation of other objects. Many perceptual studies suggest a specialised brain system for face processing [26], consistent with specificity. In addition, processing of one’s own face may involve a specialised network not used, or used to a lesser extent, for processing of other faces [3]. Comparisons between perception of faces and of non-face objects generally focus on neural *processes*, reflecting the difficulty of comparing the *content* of information represented [27].

For these reasons, it remains unclear if our effects are specific to representations of one’s own face. However, the bias towards short and wide face representation recalls similar biases for hands [11] and body shape [10]. The literature on visual perception and memory for shape do not suggest similar distortions for other objects. For example, people robustly overestimate vertical visual distances compared to horizontal distances [28], whereas we found a striking 27.7% underestimation of face height with relatively unbiased representation of face width (Figure 1). A previous study reported systematic overestimates of one’s own head size [29]. However, this conclusion was based on drawing outlines rather than locating features, and more specific analyses identified primarily width overestimation rather than height overestimation [30]. Classic studies of memory for feature locations report several Gestalt-type distortions of spatial representation, but do not mention distortions of aspect ratio [31]. The extensive literature on memory representations for complex figures [32] scarcely mentions distortions of shape – yet it seems unlikely that bias and variability as striking as those we have found for face representation would simply be overlooked. Therefore, we tentatively suggest that the effects reported here may be face-specific, but more research is needed.

### Alternative explanations

Could the factor structure we identified arise artefactually, from some process other than face representation? One possibility is a simple rotational error. Any head tilt in the facial photographs we used to measure judgement accuracy, or in the internal representation of the face that participants used to locate features, would produce systematic errors in judging the features of positions. The misrepresentation of face shape cannot be explained in this way because shape is invariant under rotation. However, some of the other distortions we noted could potentially be due to rotation. Tilt of the head (canting) is particularly likely [33], and is known to influence face recognition [34]. The pattern of errors would depend on the precise centre of rotation. For example, a tilt of the head around the centre of the face would cause equal and opposite X shifts in the hairline and chin. Crucially, our analyses would place these shifts in the same factor, with equal and opposite loadings, because the two shifts are perfectly correlated. In fact, we found that hairline and chin shifts were associated with orthogonal factors. Therefore errors in feature judgements do not appear to be due to face rotation.

A second alternative explanation would involve the spatial distribution of pointing errors around the fixation/anchor point. For example, regression to the mean might cause people to judge all facial features as closer to the nose-tip anchor point than their true location. On this account, errors should vary strictly geometrically with each feature’s position in the face, but we found several aspects of face representation that were feature-specific and independent of position in the face or on the screen. For example, we found that errors in localising the bridge of the nose were lower than errors in localising the edges of the mouth (Figure 1), even though both are approximately equidistant from the nose-tip anchor. Our factor analyses confirmed that individual features make distinct contributions to face representation, which are not simply explained by the feature’s location within the face. For example, factor Y2 loaded strongly on the mouth, but much less on the nose edges and chin, even though these features are all close together. Further, simple geometric features of our response method cannot readily explain the strong correlations between factors underlying vertical and horizontal errors. In a previous study of hand representation, patterns of distortion were shown to be invariant when the hand was presented rotated by 90 degrees relative to the body. This suggested the distortion arose from an allocentric representation of the hand, rather than from egocentric or screen-based responding. Such tests can rule out response-specific explanations of bodily distortions for the hand. Such a test is more challenging for face representation, because the face cannot be repositioned within egocentric space in the same way as the hand.

### Limitations

Finally, we acknowledge several limitations of our study. First, the number of participants is small, though it meets standards for exploratory factor analysis based on detailed simulation studies [12]. Second, our data reduction method enforced symmetry of the face around the midline, so is insensitive to possible asymmetries in representation of lateral face structures. Fluctuating asymmetry is an important facial cue to health, genetic quality, and judgements of attractiveness [35]. Future research should examine facial symmetry systematically by testing larger groups, and by directly comparing laterally inverted (mirror) versus confrontational (photograph) representations of the face [36]. Interestingly, we nevertheless identified factors involving midline shifts, confirming that asymmetry is an important aspect of face representation. Third, we have tested location judgement relative to just one central anchor, the tip of the nose. Using another anchor might, in principle, give different results – although tests of body image were largely unaffected by moving the anchor from the head to the feet [10]. Fourth, we tested only the representation of one’s own face, so we cannot say whether comparable distortions exist for less familiar faces of others, or for faces as a general semantic category. Fifth and finally, we have used factor analysis to identify the general structure of face representations from individual participants’ errors. However, we could not investigate how differences *between* individuals may influence their face representation, due to limited sample size. In particular, an individual’s face representation might depend on their actual facial structure, on their gender, or on cultural factors such as a desire to play down unusual or “unattractive” features.

## Supporting Information

Table S1 **Correlation matrix for horizontal errors in feature localisation.**
(DOCX)

Table S2 **Correlation matrix for vertical errors in feature localisation.**
(DOCX)

Figure S1 **Results of factor analysis of the face image task reveal principal factors of horizontal and vertical distortion in face representation, rendered on an average female face.** Vector show the principal feature loadings (>0.4 or <-0.4) of each factor. The vector lengths are shown at 4x the actual values for visual clarity. The percentage variance and tentative interpretation of each factor are given.
(TIF)

Figure S2 **Pictorial representation of the principal factors of horizontal and vertical distortion.** For each factor, the upper row shows an average male face distorted by a positive score of 1 standard deviation, and the bottom row shows the same face distorted by a negative unit score. Only features with high (>0.4 or <-0.4) loadings on the relevant factor were used to render the distortions.
(TIF)
