# Supplementary material for: Does My Face FIT?: A Face Image Task Reveals Structure and Distortions of Facial Feature Representation
Source: PLoS One. 2013 Oct 9;8(10):e76805. doi: 10.1371/journal.pone.0076805 (PMC3793930; doi:10.1371/journal.pone.0076805)
Supplement: Table S1 — Correlation matrix for horizontal errors in feature localisation. (DOCX) [file pone.0076805.s001.docx]

Table S1

| Hairline | 1.0000 |  |  |  |  |  |  |
| --- | --- | --- | --- | --- | --- | --- | --- |
| Chin | -0.0440 | 1.0000 |  |  |  |  |  |
| Ear | -0.0561 | -0.0906 | 1.0000 |  |  |  |  |
| Nose bridge | 0.6839 | 0.0502 | -0.1945 | 1.0000 |  |  |  |
| Nose edge | 0.1583 | 0.0457 | 0.2980 | 0.0366 | 1.0000 |  |  |
| Mouth | -0.1171 | 0.1189 | 0.5617 | -0.1107 | 0.5069 | 1.0000 |  |
| Eye | -0.1238 | 0.0195 | 0.7027 | -0.1635 | 0.4246 | 0.8085 | 1.0000 |
|  | Hairline | Chin | Ear | Nose bridge | Nose edge | Mouth | Eye |

Table S1. Correlation matrix for horizontal errors in feature localisation
