# Supplementary material for: Does My Face FIT?: A Face Image Task Reveals Structure and Distortions of Facial Feature Representation
Source: PLoS One. 2013 Oct 9;8(10):e76805. doi: 10.1371/journal.pone.0076805 (PMC3793930; doi:10.1371/journal.pone.0076805)
Supplement: Table S2 — Correlation matrix for vertical errors in feature localisation. (DOCX) [file pone.0076805.s002.docx]

Table S2

| Hairline | 1.0000 |  |  |  |  |  |  |
| --- | --- | --- | --- | --- | --- | --- | --- |
| Chin | -0.4810 | 1.0000 |  |  |  |  |  |
| Ear | 0.1745 | 0.0123 | 1.0000 |  |  |  |  |
| Nose bridge | 0.6928 | -0.4304 | 0.2000 | 1.0000 |  |  |  |
| Nose edge | 0.0811 | 0.3254 | 0.0165 | -0.0109 | 1.0000 |  |  |
| Mouth | -0.2733 | 0.7768 | 0.1487 | -0.2501 | 0.5842 | 1.0000 |  |
| Eye | 0.7149 | -0.3836 | 0.1689 | 0.8265 | 0.1694 | -0.2021 | 1.0000 |
|  | Hairline | Chin | Ear | Nose bridge | Nose edge | Mouth | Eye |

Table S2. Correlation matrix for vertical errors in feature localisation.
