# Supplementary figures and images for: Does My Face FIT?: A Face Image Task Reveals Structure and Distortions of Facial Feature Representation
Source: PLoS One. 2013 Oct 9;8(10):e76805. doi: 10.1371/journal.pone.0076805 (PMC3793930; doi:10.1371/journal.pone.0076805)

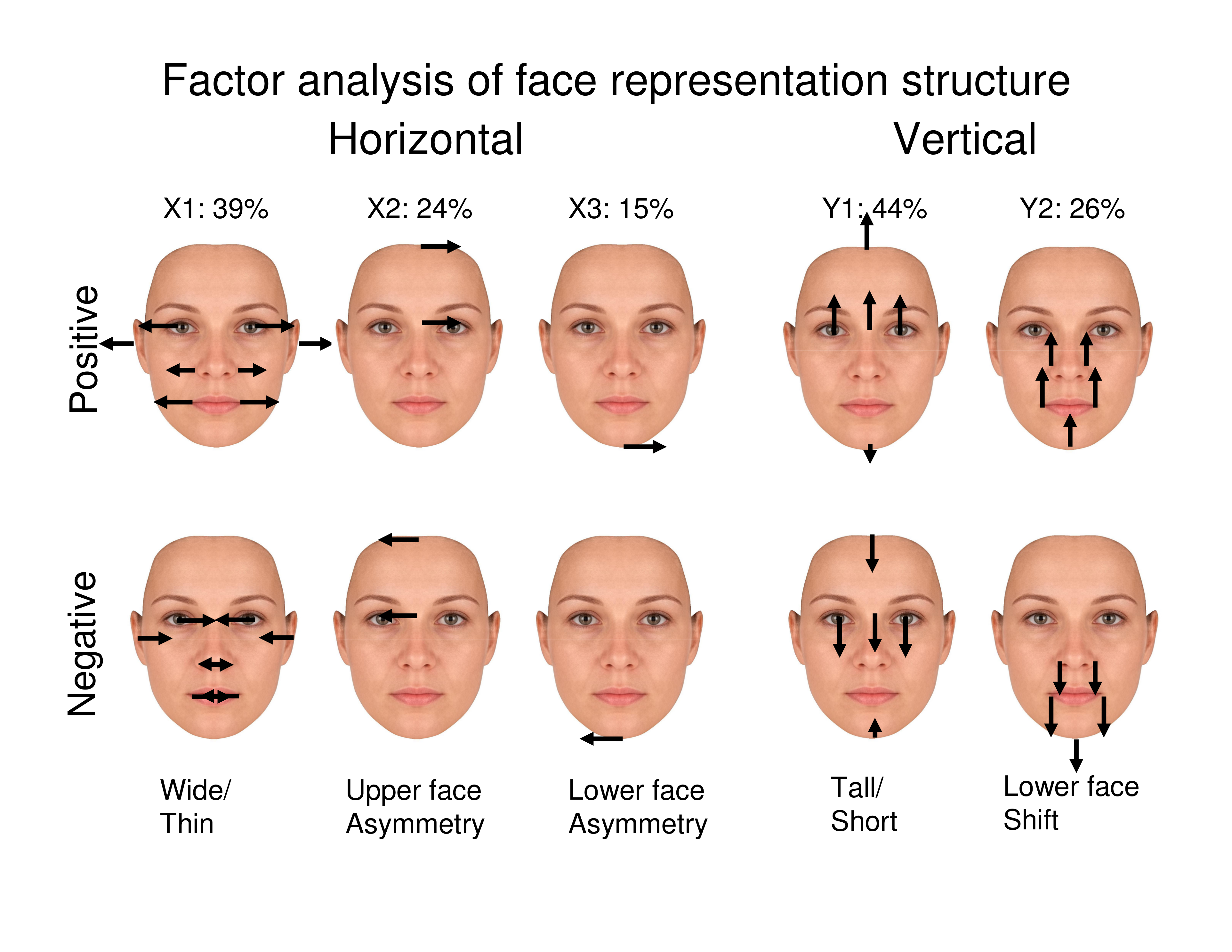

Supplement: Figure S1 — Results of factor analysis of the face image task reveal principal factors of horizontal and vertical distortion in face representation, rendered on an average female face. Vector show the principal feature loadings (>0.4 or <-0.4) of each factor. The vector lengths are shown at 4x the actual values for visual clarity. The percentage variance and tentative interpretation of each factor are given. (TIF) [file pone.0076805.s003.tif]

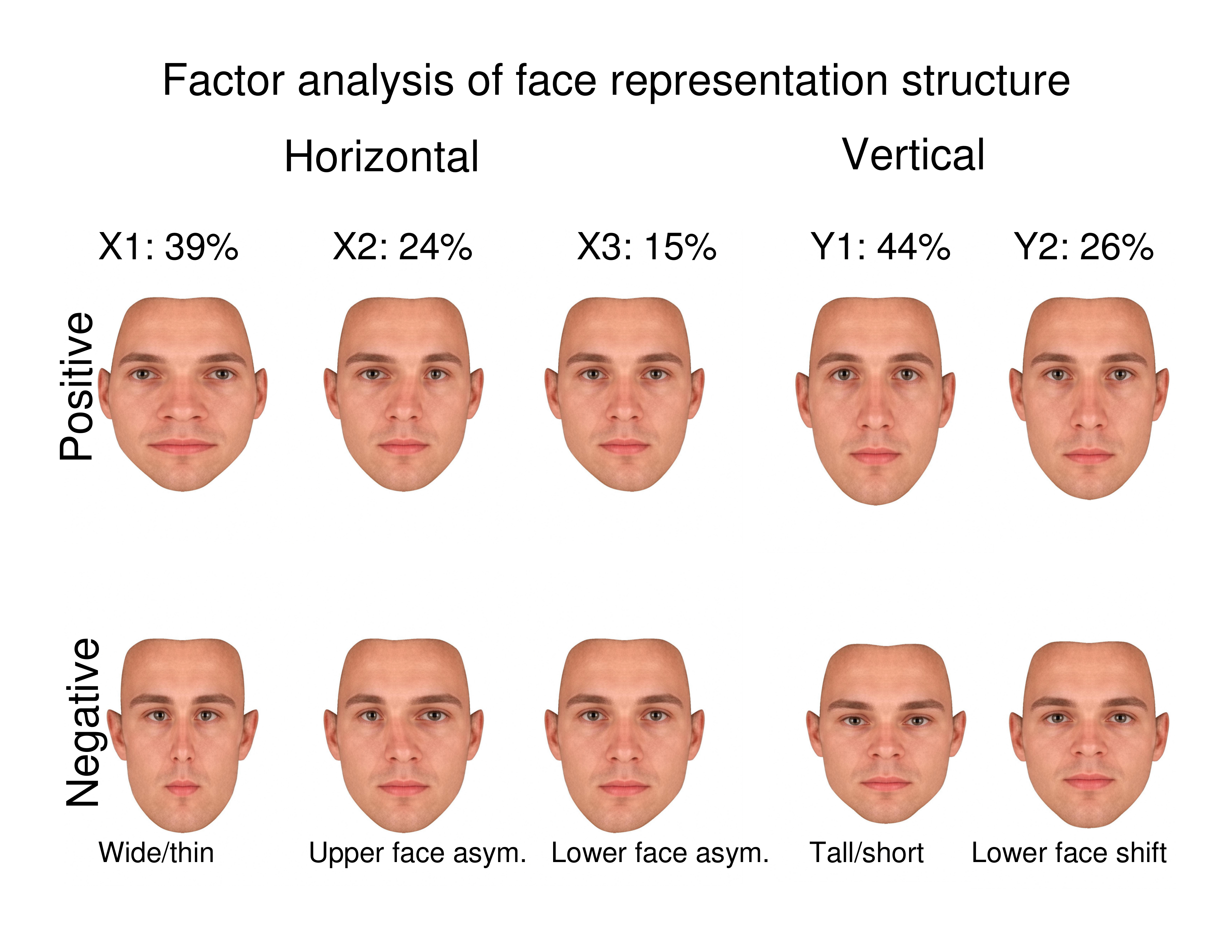

Supplement: Figure S2 — Pictorial representation of the principal factors of horizontal and vertical distortion. For each factor, the upper row shows an average male face distorted by a positive score of 1 standard deviation, and the bottom row shows the same face distorted by a negative unit score. Only features with high (>0.4 or <-0.4) loadings on the relevant factor were used to render the distortions. (TIF) [file pone.0076805.s004.tif]
